# HIV viral protein expression disrupts bone tissue homeostasis in mice

**DOI:** 10.3389/fendo.2026.1920538

**Published:** 2026-10-20

**Authors:** Joan Villarroya, Natalia Artigas, Tania Quesada-López, Rubén Cereijo, Pere Domingo, Francesc Villarroya, Francesc Ventura, Marta Giralt

**Affiliations:** 1 Department of Biochemistry and Molecular Biomedicine and Institute of Biomedicine (IBUB), University of Barcelona, Barcelona, Spain; 2 CIBER “Fisiopatología de la Obesidad y Nutrición” (CIBEROBN), Instituto de Salud Carlos III, Madrid, Spain; 3 Department of Physiological Sciences, University of Barcelona, and IDIBELL, Barcelona, Spain; 4 Infectious Diseases Unit, Hospital de la Santa Creu i Sant Pau, Barcelona, Spain

**Keywords:** adipose tissue, animal model, bone, HIV, metabolism

## Abstract

HIV infection is associated with metabolic and skeletal comorbidities that persist despite effective antiretroviral therapy. However, the direct contribution of viral proteins to these alterations remains incompletely understood. Here, we investigated the impact of HIV transgene expression on adipose tissue content, inflammatory status, and bone tissue homeostasis in mice. HIV transgenic mice Tg26 exhibited lipoatrophy in both young and aged animals, accompanied by only mild changes in systemic inflammatory parameters. In parallel, HIV transgene expression was associated with early-onset bone loss, affecting both cortical and trabecular compartments. Molecular analyses of calvarial bone revealed dysregulation of key regulators of bone remodeling, indicating an imbalance in osteoblast and osteoclast activity. Consistently, circulating bone turnover markers were elevated in HIV transgenic mice, supporting a state of increased, coupled high-turnover bone remodeling. Collectively, these findings suggest that HIV viral protein expression is sufficient to induce persistent alterations in skeletal homeostasis, highlighting a role of viral factors in the pathogenesis of HIV-associated metabolic and bone disease.

## Introduction

Bone health is increasingly recognized as a major clinical concern in people living with HIV (PWH). Since the introduction of combination antiretroviral therapy (ART), HIV infection has evolved from a rapidly progressive fatal disease into a chronic manageable condition, resulting in a substantial increase in life expectancy among PWH. Consequently, the clinical spectrum of HIV-associated morbidity has shifted from opportunistic infections and AIDS-defining illnesses toward chronic non-communicable diseases, including cardiovascular disease, metabolic disorders, neurocognitive impairment, and skeletal complications (1). Among these, osteoporosis and fragility fractures have emerged as important causes of disability, reduced quality of life, and healthcare burden (2, 3). Importantly, epidemiological studies consistently demonstrate that PWH exhibit a higher prevalence of reduced bone mineral density (BMD), osteopenia, osteoporosis, and fracture risk compared with HIV-negative populations (4–6). This increased susceptibility is observed across different age groups, including relatively young individuals, and persists even in patients receiving suppressive ART, suggesting that both HIV infection itself and its treatment contribute to skeletal deterioration.

Bone is a highly dynamic tissue that undergoes continuous remodeling through the coordinated actions of bone-forming osteoblasts and bone-resorbing osteoclasts. Maintenance of skeletal integrity requires a tightly regulated balance between these cellular populations. In the context of HIV infection, multiple mechanisms converge to disrupt bone homeostasis and favor net bone resorption. The pathogenesis of HIV-associated bone disease is therefore considered multifactorial, involving direct viral effects, chronic inflammation and immune dysregulation, antiretroviral drug toxicity, endocrine alterations, nutritional deficiencies, and lifestyle-related factors (7). Some experimental studies have shown direct effects of HIV infection and/or of some of the HIV proteins on bone cell function by stimulating osteoclast differentiation and activity while simultaneously impairing osteoblast proliferation, differentiation, and survival (8). These alterations ultimately compromise bone formation and accelerate skeletal loss.

In parallel, chronic immune activation and persistent low-grade inflammation are recognized hallmarks of HIV infection, even under effective viral suppression. Elevated circulating levels of pro-inflammatory cytokines such as tumor necrosis factor-alpha (TNF-α), interleukin-1 beta (IL-1β), interleukin-6 (IL-6), and receptor activator of nuclear factor kappa-B ligand (RANKL) contribute to enhanced osteoclastogenesis and increased bone turnover (9). The RANK/RANKL/osteoprotegerin (OPG) signaling pathway, which constitutes a central regulatory axis in bone remodeling, appears to be profoundly altered during HIV infection (10, 11). Increased RANKL expression together with reduced OPG levels promotes osteoclast activation and bone resorption, thereby contributing to progressive skeletal fragility. Additionally, HIV-associated immune senescence and alterations in T-cell and B-cell function further exacerbate disturbances in bone metabolism (12).

Although ART has dramatically improved survival and reduced HIV-related mortality, antiretroviral drugs may independently contribute to bone loss. Initiation of ART is typically associated with a rapid decline in BMD during the first one to two years of treatment, with reported reductions ranging from 2% to 6%, regardless of the regimen employed (13). However, certain antiretroviral agents have been associated with a greater detrimental impact on bone health. In particular, tenofovir disoproxil fumarate (TDF) has been strongly linked to reductions in BMD and increased bone turnover (14). Proposed mechanisms include renal phosphate wasting, mitochondrial toxicity, oxidative stress, alterations in vitamin D metabolism, and secondary hyperparathyroidism (15). Protease inhibitors and some integrase strand transfer inhibitors have also been implicated in metabolic and endocrine disturbances that may negatively affect skeletal homeostasis (2). Thus, while ART remains indispensable for HIV management, long-term treatment may contribute to the development of osteopenia and osteoporosis.

Beyond viral and treatment-related factors, PWH frequently present with additional comorbidities and lifestyle factors that further increase the risk of skeletal disease. Co-infections such as hepatitis B virus (HBV) and hepatitis C virus (HCV), smoking, excessive alcohol consumption, low body mass index, physical inactivity, hypogonadism, malnutrition, and vitamin D deficiency are highly prevalent in this population and are all independently associated with impaired bone health (3, 16). Socioeconomic determinants and disparities in healthcare access may additionally influence osteoporosis screening, diagnosis, and treatment in PWH. More recently, growing attention has focused on the role of the gut microbiota in HIV-associated chronic inflammation and metabolic dysfunction. HIV infection is associated with intestinal barrier disruption and microbial translocation, which may perpetuate systemic immune activation and contribute to bone loss through inflammatory and immunometabolic pathways (17).

Despite increasing recognition of HIV-associated bone disease, the precise molecular and cellular mechanisms underlying skeletal alterations remain incompletely understood. Animal models represent valuable tools to investigate the direct pathogenic effects of HIV proteins independently of confounding clinical variables commonly present in human cohorts. Several HIV-1 transgenic rodent models have been developed to study the deleterious effects of viral gene expression in different tissues and organ systems (18). Among these, the heterozygous HIV-1 transgenic mouse model Tg26 has been widely used to investigate HIV-associated pathophysiological alterations because it constitutively expresses multiple HIV-1 viral proteins in the absence of productive viral replication (19). In the present study, we used both young and adult Tg26 mice to explore alterations in skeletal tissue associated with HIV-1 infection.

## Materials and methods

### Animal experiments

FVB/N-Tg(HIV)26Aln/PkltJ (Tg26) FVB male mice (Jackson Laboratories) and wild-type littermates were maintained 6 or 25 weeks at standard animal facility conditions (12-h dark/light cycle with *ad libitum* access to food and water) until sacrificed by decapitation. Blood and tissues were collected. Adipose tissues were dissected and weighted. Skeletal tissues were dissected and processed for further analyses: calvaria were ground to powder for RNA extraction and femurs were fixed (see below). All experiments were performed in accordance with European Community Council directive 86/609/EEC and the experiments and numbers of animals to be used were approved by the Institutional Animal Care and Use Committee of the University of Barcelona.

### Bone morphometric microCT analysis

High-resolution images from fixed femurs (4% PFA, 24 h at 4 °C), were acquired using a computerized microtomography imaging system (Skyscan 1076, Bruker microCT) in accordance with the recommendations of the American Society of Bone and Mineral Research (ASBMR) (20). Samples were scanned in air at 49kV and a power of 200 μA with an exposure time of 800 ms, using a 1 mm aluminium filter and an isotropic voxel size of 11 μm. Two-dimensional images were obtained every 1° of a 180° rotation. Images were reconstructed using the NRecon software (version 1.7.1.0, Bruker) and analysed with a CT Analyzer (version 1.17.7.2, SkyScan). For trabecular measurements, manual ROIs were selected by contouring the trabecular compartment and excluding cortical bone, starting 0.6 mm from the distal growth plate of the femur and extending to the diaphysis for 2 mm. A fixed offset was used for both age groups. For cortical parameters, we analyzed a 3-mm section of the mid-diaphysis. Trabecular and cortical ROIs were represented using CT-Vol (version 2.3.2.0, SkyScan).

### Serum biochemistry

Glucose and triglyceride levels were measured in blood using Accutrend Technology (Roche Diagnostics). FGF21 (RD291108200R, Biovendor), CTX-1, and P1NP EIA (Immunodiagnosticsystems) levels were quantified by ELISA. Insulin, adipokines, cytokines and bone biomarkers were quantified using a Multiplex system (MADKMAG-HK, MBNMAG-41K, Millipore).

### RNA isolation and real-time quantitative PCR

RNA was extracted from calvaria using a NucleoSpin RNA kit (Macherey-Nagel), and the mRNA levels were determined by quantitative reverse transcription-polymerase chain reaction (qRT-PCR), using the appropriate, TaqMan assays (Applied Biosystems) (Rankl/Tnfsf11, Mm00441906_m1; Opg/Tnfrsf11b, Mm00435454_m1; Fgf23, Mm00445621_m1; Sost, Mm00470479_m1; Col1a1, Mm00801666_g1; Dkk1, Mm00438422_m1; Sp7, Mm04933803_m1; Runx2, Mm00501584_m1; Postn, Mm01284919_m1; Mmp13, Mm00439491_m1; Spp1, Mm00436767_m1). The mRNA level of each gene of interest was normalized to that of a housekeeping reference gene (Tbp, Mm01277042_m1) using the comparative (2^-ΔCt^) method.

### Statistical analysis

Statistical significance was assessed using the two-way ANOVA followed by the Tukey’s *post-hoc* tests, which were applied with the GraphPad statistical software (GraphPad 8-Prism Software). Outlier values were detected using the Grubbs test and excluded from the analyses following its established significance threshold. Statistical significance was set with an α-value of p<0.05, and the underlying assumptions for validity were assessed for all tests. Data are shown as means ± s.e.m.

## Results

### HIV transgenic mice show lipoatrophy in young and aged mice, with mild affection of inflammatory parameters

We first analyzed the expression of the HIV transgene in Tg26 heterozygous (Tg26+/-) mice by measuring Tat mRNA content in several tissues, as Tat mRNA serves as a marker of HIV expression and reflects active viral transcription. Tat mRNA was substantially expressed in all tissues analyzed from Tg26+/- mice, particularly in inguinal (iWAT) and epididymal (eWAT) white adipose tissues and bone (**Supplementary Figure 1**). Young Tg26+/- transgenic mice (6-week-old) exhibited reduced body weight compared with controls, and this difference persisted into adulthood (25-week-old mice). This reduction was associated with decreased adiposity, while tibia length and food intake remained unchanged. The amounts of subcutaneous (iWAT) and visceral (eWAT) depots, but not liver, were lower in Tg26+/- mice compared to wild type littermates (**Table 1**). Glucose homeostasis parameters (glycemia, insulinemia) were unaltered. Serum pro-inflammatory cytokine levels (e.g., Interleukin 6, PAI-1 total) showed a tendency to be increased in Tg26+/- mice, especially at the 25-week-old setting, whereas leptin levels were dramatically reduced at both ages. Other inflammation marker cytokines, such as TNFα, were also found significantly increased in 6-week-old mice (**Table 1**).

**Table 1:** Morphological, metabolic, endocrine and inflammation-related parameters in Tg26 heterozygous mice (Tg26+/-) and wild-type littermates (values are mean ± sem).

	*6-week old*		*25-week old*		*p-value*
	WT	Tg26+/-	WT	Tg26+/-	
Body weight (g)	23.24 ± 0.35	21.31 ± 0.35**	36.70 ± 0.63	33.83 ± 0.40**	p ≤ 0.0001, genotype
Tibia length (mm)	16.16 ± 0.35	15.53 ± 0.33	18.60 ± 0.10	18.71 ± 0.14	
Food intake (kcal/animal/day)	14.85 ± 2.19	15.93 ± 3.23	12.20 ± 1.49	11.64 ± 1.46	
Tissue weight (mg)					
iWAT	181.2 ± 7.0	133.50 ± 9.81	534.00 ± 28.06	335.80 ± 32.00****	p ≤ 0.0001, genotype
eWAT	246.5 ± 27.3	151.70 ± 16.42	983.2 ± 30.56	564.40 ± 111.20***	p=0.0001, genotype
Liver	1306 ± 44	1517 ± 62	1478 ± 62	1485 ± 51	
Circulating parameters					
Glucose (mg/dL)	190.6 ± 11.1	184.3 ± 6.1	178.10 ± 13.23	188.40 ± 9.02	
Insulin (pg/mL)	219.80 ± 38.41	325.30 ± 93.15	1010 ± 229.70	792.30 ± 139.90	
Triglycerides (mg/dL)	251 ± 12.9	328.20 ± 32.79	280.00 ± 29.51	234.20 ± 20.96	
Leptin (pg/mL)	132.8 ± 48.1	75.21 ± 31.29	3248 ± 944.00	1378 ± 380.50*	p=0.041, genotype
MCP1 (pg/mL)	30.4 ± 8.8	42.35 ± 7.33	2706 ± 965	1008 ± 619	
PAI-1 total (pg/mL)	3179 ± 888	1456 ± 343	3620 ± 248	5141 ± 731	
Resistin (pg/mL)	192.9 ± 38.5	259.9 ± 40.65	625.2 ± 67.01	780.1 ± 148.7	
Interleukin 6 (pg/mL)	10.37 ± 2.04	23.95 ± 7.82	7.91 ± 2.43	19.23 ± 8.74	
TNFα (pg/mL)	3.63 ± 1.50	13.48 ± 2.52**	ND	ND	p=0.006, (One-way)

Two-way ANOVA significance test (p-value) is shown in the table. *p<0.05; **p<0.01; ***p<0.001; ****p<0.0001 for *post-hoc* Tukey’s test between genotypes.

iWAT, inguinal white adipose tissue; eWAT, epididymal white adipose tissue; MCP1, monocyte chemoattractant protein 1; PAI-1, plasminogen activator inhibitor type 1; TNFα, tumor necrosis factor alpha. ND, Not detected.

### HIV transgene expression in mice is associated with early bone loss

Measurements of tibial length and cortical cross-sectional perimeter revealed little evidence that transgene expression affected overall bone development in heterozygous Tg26 mice (**Table 1**; **Figure 1A**). However, multiple cortical and trabecular bone parameters in the femur were significantly decreased in transgenic mice, indicating impaired bone homeostasis and a bone loss phenotype. For instance, microCT revealed a significant decrease in cortical bone volume and thickness in femur from young and old Tg26 heterozygous mice. Data from trabecular parameters confirmed the bone loss phenotype. Tg26+/- mice showed a decrease in trabecular thickness and number, and an increase in trabecular separation, as revealed by significant genotype effects. The percentage of bone volume fraction, another indicative of mineralized bone per unit volume of the sample, was also decreased in transgenic mice (**Figure 1B**).

**Figure 1:**
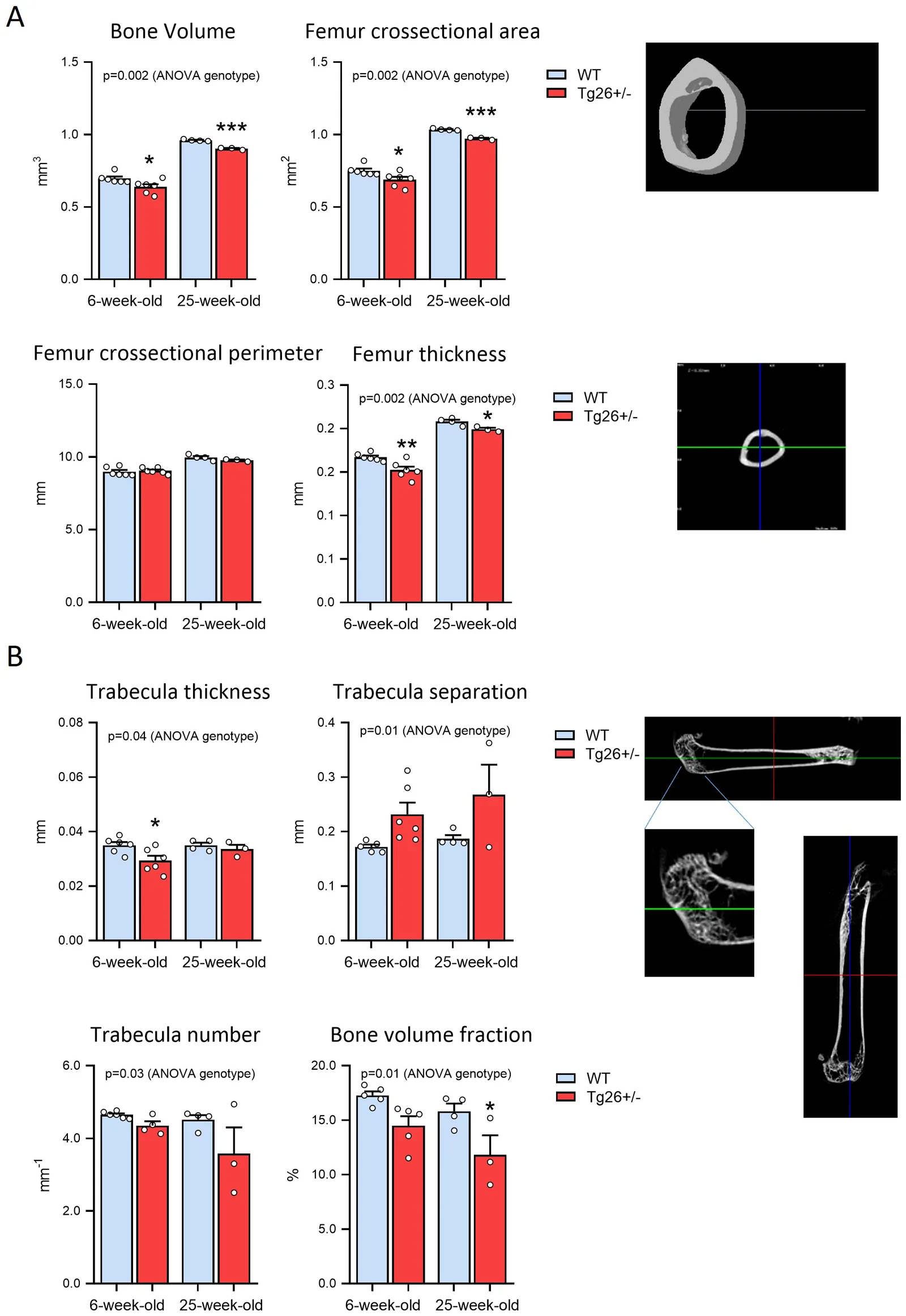
Bone morphometric parameters in Tg26 heterozygous mice (Tg26+/-) and wild-type controls (WT). Measurement of selected parameters related to bone physiology in femur **(A)** and trabeculas **(B)** from 6 and 25-week-old mice (n=3-6). The bars represent means ± s.e.m. Two-way ANOVA significance test (p) is shown in the graph. No significant genotype-by-age interactions were detected for any of the evaluated parameters. *p<0.05; **p<0.01; ***p<0.001, for *post-hoc* Tukey’s test between genotypes.

### Gene expression in calvaria indicate an alteration in markers of bone turnover

Gene expression in calvaria bone showed a general profile indicating enhanced expression of inhibitors of bone formation, such as the RANK ligand mRNA (*Rankl*) and sclerostin transcript (*Sost*), especially in 25-week mice (**Figure 2**). *Runx2* expression exhibited a significant genotype-by-age interaction, suggesting altered osteoblast differentiation and impaired early bone formation in transgenic mice. Expression of *Mmp13* (matrix metalloproteinase-13), a key mediator of extracellular matrix remodeling during bone turnover, was reduced in Tg26+/- mice. Significant genotype-by-age interactions were also observed for *Dkk1* (Dickkopf-related protein 1) and *Postn* (periostin), indicating dysregulated bone remodeling pathways in the transgenic mice. No changes were found when measuring other bone physiological markers such as *Fgf23*, *Col1a1, Spp1* and Osterix (also known as *Sp7*) mRNA expression (**Figure 2**).

**Figure 2:**
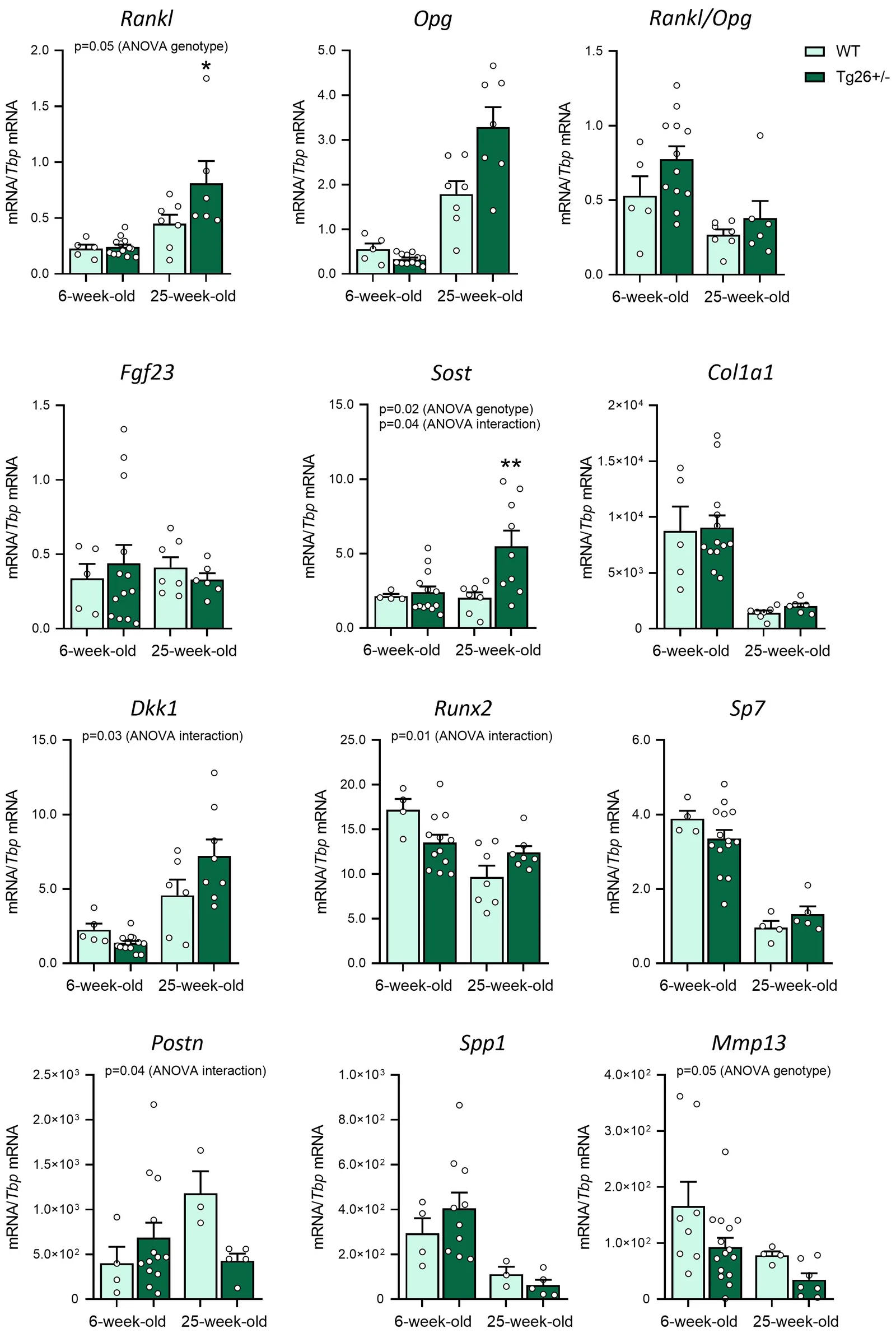
Gene transcript expression in bone from Tg26 heterozygous mice (Tg26+/-) mice and wild-type controls (WT). Measurement of mRNA markers of bone turnover in calvaria from 6 and 25-week-old mice (n=4-13). The bars represent means ± s.e.m. Two-way ANOVA significance test (p) is shown in the graph. Interaction means genotype-age interaction. *p<0.05; **p<0.01, for *post-hoc* Tukey’s test between genotypes.

### Circulating bone turnover markers are increased in HIV transgenic mice

We also measured a subset of circulating proteins that evaluate the osteoclast/osteoblast activity and bone turnover in rodents. Consistent with the findings described above, circulating CTX-1 levels, a marker of osteoclast-mediated bone resorption, were elevated in transgenic mice. Likewise, circulating P1NP levels, a marker of osteoblast activity and bone formation, showed a tendency to increase in 25-week-old transgenic mice. Together, these findings are indicative of enhanced coupled high-turnover bone remodeling, characterized by concomitant increases in both bone resorption and bone formation. In addition, circulating levels of osteoprotegerin (OPG), a key anti-osteoclastogenic factor that inhibits RANKL signaling, were also elevated. None of the other circulating bone biomarkers assessed showed significant changes (**Table 2**).

**Table 2:** Bone homeostasis-related factors in plasma from Tg26 heterozygous mice (Tg26+/-) and wild-type littermates (values are mean ± sem).

	*6-week old*		*25-week old*		*p-value*
	WT	Tg26+/-	WT	Tg26+/-	
FGF21 (pg/mL)	465.2 ± 68.9	1105 ± 202.7	410.5 ± 110.9	382.4 ± 81.51	
FGF23 (pg/mL)	312.8 ± 26.5	342.3 ± 39.90	230.0 ± 23.03	241.5 ± 27.47	
SOST (pg/mL)	74.9 ± 6.3	83.29 ± 5.39	86.74 ± 3.00	75.62 ± 9.93	
ACTH (pg/mL)	103.3 ± 30.1	95.08 ± 14.20	53.74 ± 15.46	53.71 ± 17.80	
OPG (pg/mL)	1423 ± 100	1527 ± 115	1208 ± 69.90	1818 ± 240*	p=0.02, genotype
DKK1 (pg/mL)	4491 ± 412	5736 ± 661	8923 ± 1448	9912 ± 1816	
CTX-1 (ng/mL)	40.7± 2.6	48.23 ± 3.35	12.56 ± 1.46	21.13 ± 3.67*	p=0.05, genotype
P1NP EIA (ng/mL)	4.96± 0.41	4.96 ± 0.416	1.074 ± 0.073	1.667 ± 0.162	

Two-way ANOVA significance test (p-value) is shown in the table. *p<0.05 for *post-hoc* Tukey’s test between genotypes.

FGF21, Fibroblast Growth Factor 21; FGF23, Fibroblast Growth Factor 23; SOST, Sclerostin; ACTH, Corticotropin; OPG, Osteoprotegerin; DKK1, Dickkopf-related protein 1; CTX-1, Carboxy-terminal collagen crosslinks (C-terminal telopeptide); P1NP EIA, N-terminal propeptide of type I procollagen.

## Discussion

The widespread use of combination ART has transformed HIV infection into a chronic condition, markedly improving life expectancy and shifting clinical priorities toward the management of long-term comorbidities. As a result, non-communicable diseases including cardiovascular disease, metabolic dysfunction, chronic kidney disease, neurocognitive impairment, and skeletal disorders, now represent major contributors to morbidity and mortality in this population (21). Among these conditions, alterations in bone metabolism are particularly relevant due to their high prevalence and impact on quality of life. PWH have an increased risk of reduced BMD, osteopenia, osteoporosis, and fragility fractures compared with HIV-negative individuals (22). Notably, even PWH who spontaneously control viral replication and do not require ART (long-term non-progressors) exhibit skeletal alterations, suggesting that HIV infection itself contributes to impaired bone health (23). These abnormalities are multifactorial, arising from a complex interplay among traditional risk factors, persistent immune activation and inflammation, antiretroviral therapy, and metabolic and lifestyle influences. Thus, and in the context of an increasingly aging HIV population, preserving bone health has become a critical component of long-term clinical management and an important focus of current clinical and translational research.

The present study aimed to provide further insight into the mechanisms underlying HIV-associated bone alterations by using a preclinical mouse model. Unlike clinical studies in PWH, where the effects of HIV infection are often difficult to disentangle from those of ART, viral suppression status, aging, and other comorbidities, the Tg26 mouse constitutes a valuable model to examine the intrinsic contribution of viral factors to pathogenesis. Here, we experimentally investigated whether bone alterations are directly driven by HIV-derived proteins, independently of ART exposure and viral load.

Present findings in young and adult Tg26 mice showing lower body weight associated with a selective reduction in subcutaneous and visceral white adipose tissue depots, while tibia length, liver mass, and food intake remained unchanged, are consistent with previous studies in Tg26 mice reporting reduced adiposity and altered adipose tissue function as a consequence of HIV-protein expression. In particular, earlier work described a reduction in visceral adipose tissue and impaired adipokine production (24), whereas a more recent study has demonstrated decreases in both visceral and subcutaneous fat mass without changes in food intake (25). Together, these observations support the notion that HIV-derived proteins contribute to alterations in body composition independently of ART. This is also consistent with studies analyzing the relative contributions of HIV-infection and ART to adipose tissue gene expression in PWH and showing that some disturbances are already present in untreated individuals (26).

Despite the marked reduction in adiposity, Tg26 mice displayed normal glycemia and insulinemia, indicating preserved glucose homeostasis. In contrast, these mice exhibited evidence of low-grade systemic inflammation, with increased TNFα levels at 6 weeks of age and a tendency toward higher IL-6 and PAI-1 concentrations at 25 weeks. Similar inflammatory alterations have been previously reported in Tg26 mice, including increased TNFα and IL-6 expression (24), supporting a direct role of HIV viral proteins in promoting chronic low-grade inflammation. As expected from the reduced white adipose tissue mass, circulating leptin levels were markedly decreased at both ages, consistent with previous observations in this model (24). Given the recognized effects of inflammatory cytokines and leptin on bone remodeling, these alterations may contribute to the skeletal phenotype observed in Tg26 mice.

Consistently with the high prevalence of low bone mineral density and osteoporosis reported in PWH (2, 3, 27), microCT analysis revealed a marked deterioration of both cortical and trabecular bone compartments in Tg26 mice. Cortical bone volume, and thickness were significantly reduced in both young and older animals, whereas femoral cross-sectional perimeter remained unchanged, indicating preserved skeletal development but impaired bone acquisition and maintenance. Trabecular bone was similarly affected, with reductions in trabecular thickness, trabecular number, and bone volume fraction (BV/TV), together with increased trabecular separation. Importantly, these morphometric alterations were already evident in young mice, suggesting that bone loss is initiated early and persists with aging, with more evident changes observed in cortical bone. In combination with the inflammatory profile observed in these animals, our results also suggest that chronic HIV protein expression is sufficient to disrupt normal bone remodeling.

Alterations in calvarial bone gene expression observed in Tg26 mice provide mechanistic support for the skeletal phenotype identified by microCT. Increased expression of *Rankl*, and *Sost* indicates a bone microenvironment favoring resorption while suppressing bone formation. RANKL is a key regulator of osteoclast differentiation and activity, whereas sclerostin is a potent inhibitor of Wnt/β-catenin signaling, a pathway essential for osteoblast differentiation and bone formation (28, 29). We also observed reduced *Mmp13* and *Postn* expression in aged transgenic mice, which may indicate subtle age-related changes in bone remodeling and extracellular matrix homeostasis. However, the expression of *Spp1*, *Col1a1* and *Fgf23* remained unchanged, while only modest alterations were observed in *Runx2* expression. These findings suggest that osteoblast and osteocyte activity, as well as matrix production and phosphate homeostasis, are largely preserved in transgenic mice. In contrast, the elevated CTX-I levels observed in transgenic mice, together with a tendency toward increased P1NP levels at 25 weeks of age, indicate a high-turnover bone phenotype. This is consistent with the net bone loss indicated by the microCT phenotype of Tg26 mice, which revealed significant deficits in both cortical and trabecular bone compartments. Present findings also align with previous experimental studies showing that exposure to non-replicative HIV or directly to some HIV-derived proteins, including gp120, tat, and vpr, upregulate RANKL and reduced OPG leading to increased osteoclastogenesis (11, 30–33). We acknowledge the apparent discrepancy with our observation of elevated circulating OPG levels. However, this increase may reflect a compensatory counter-regulatory response aimed at limiting excessive RANKL-mediated osteoclast activation and bone resorption. Raynaud-Messina et al. (34) provide the first evidence that osteoclasts can serve as a reservoir for HIV, as well as demonstrating the direct deleterious effects of HIV infection on the bone resorption machinery. Recent studies in PWH indicated that HIV infection and associated inflammation disrupt bone remodeling through activation of the RANKL/OPG axis and suppression of osteoblastogenesis (35, 36). HIV1-infected macrophages, by secreting RANKL, favor the recruitment of osteoclasts, also participating in bone disorders observed in PWH (37).

Nevertheless, the bone phenotype observed could be substantially secondary to the renal disease previously reported in this model (19), rather than a direct action of viral proteins on bone cells, particularly in a progressive manner during aging (38). However, given the central role of the FGF23–kidney axis in phosphate and mineral homeostasis, if the bone phenotype in Tg26+/- mice were entirely driven by renal impairment, alterations in FGF23 would be expected. Instead, both *Fgf23* mRNA expression and serum levels remain unchanged, which makes it less likely that the bone phenotype is predominantly secondary to a major renal or mineral disturbance. Moreover, the relatively preserved expression of *Sp7, Spp1* and *Col1a1*, together with the selective changes in *Sost, Mmp13*, and *Postn*, supports a model in which bone remodeling is specifically altered in Tg26+/- mice.

Several limitations should be considered when interpreting these findings. First, confirmation of the transcriptional changes in femoral or tibial osteoblasts would help determine whether the observed molecular alterations are also present at the anatomical site where the morphometric phenotype was assessed. Second, the study included only male animals, precluding assessment of potential sex-dependent effects. The relatively small sample size in old mice also limits statistical power and the generalizability of the findings. Finally, the heterozygous transgenic model used here, which constitutively expresses a defined set of viral proteins, does not fully recapitulate natural viral infection, including differences in viral dynamics, host responses, and the broader repertoire of viral factors. Thus, these findings should be interpreted within the context and limitations of the experimental model.

In conclusion, our findings contribute to the growing body of evidence suggesting that HIV-associated skeletal abnormalities are not exclusively a consequence of antiretroviral treatment but may also arise from the biological effects of viral proteins on bone cells and the bone microenvironment. By isolating these effects, the present study provides a clearer understanding of the specific characteristics of bone dysfunction induced by HIV-related factors and helps to elucidate the mechanisms through which the virus may impair bone remodeling and skeletal integrity. Moreover, Tg26 mice provide a promising model to study how antiretroviral drugs and environmental factors influence skeletal homeostasis in the context of HIV-1 infection-related events already present within the organism. These insights may have important implications for the development of targeted therapeutic strategies aimed at preserving bone health in people living with HIV.

## Data Availability

The raw data supporting the conclusions of this article will be made available by the authors, without undue reservation.
